# Mixotrophic Phytoflagellate Bacterivory Field Measurements Strongly Biased by Standard Approaches: A Case Study

**DOI:** 10.3389/fmicb.2017.01398

**Published:** 2017-07-26

**Authors:** Ruth Anderson, Klaus Jürgens, Per J. Hansen

**Affiliations:** ^1^Marine Biological Section, Department of Biology, University of Copenhagen Helsingør, Denmark; ^2^Leibniz Institute for Baltic Sea Research Rostock, Germany

**Keywords:** mixotrophy, phytoflagellate, bacterivory, diel cycle, heterotrophic nanoflagellate

## Abstract

Bacterivory among small (≤20 μm) phytoflagellates (SP) is increasingly recognized as a globally relevant phenomenon, impacting a wide range of aspects from primary production levels to marine fisheries. However, to correctly parametrize mixotrophic SP in biogeochemical and food web models, a better understanding of the magnitude and regulation of *in situ* SP feeding is urgently needed. Current methods to determine SP bacterivory in the field may introduce biases by treating these organisms as equivalent to heterotrophic nanoflagellates (HNF). In the present case study we experimentally tested two generally employed assumptions of such studies: (A) bacterivory rates of the whole SP community and of distinct SP groups remain constant over ‘short’ time scales (hours to a day) and (B) SP community ingestion rates approximate the average ingestion rate of all feeding individuals. Food vacuole markers (acidotropic probes), were applied along the diel cycle at three stations in December 2015, and May and June 2016. In December and June, surrogate prey (fluorescently labeled bacteria) were used in parallel at one sampling station. Sampling at different times of day produced an up to fourfold difference in estimates of SP daily bacterivorous impact. In contrast, daily bacterivory estimates for HNF remained constant in almost all cases. The perceived principal SP bacterivorous groups also shifted strongly. As an example, picoeukaryotes dominated total SP bacterivory in daylight hours but completely ceased to feed at night. Finally, a large fraction of the SP community was not feeding at all time points tested. This lead to significant errors in estimated ingestion rates determined using the whole SP community, being up to 16 times lower than those determined solely for actively feeding mixotrophic SP. Overall, this case study indicates that applying the two commonly used premises outlined above can introduce significant biases and considerably alter our perception of mixotrophy in a given system.

## Introduction

Small (<20 μm) phototrophic flagellated protists [small phytoflagellates (SP)] are key contributors to marine primary production, which produces half the oxygen on the planet and drives the biological carbon pump (Falkowski et al., 1998; Worden et al., 2015). Traditionally, SP were thought to be strict phototrophs (Flynn et al., 2013). However, bacterivory among SP is increasingly recognized as a globally distributed and environmentally relevant trophic strategy employed by most, if not all, major SP phylogenetic lineages (Unrein et al., 2007; Hartmann et al., 2012; McKie-Krisberg et al., 2015). Punctual studies have shown that SP feeding can account for up to 65% of total bacterivory in the Mediterranean (Unrein et al., 2007) and up to 95% in the North Atlantic Ocean (Zubkov and Tarran, 2008). On a global scale, whether SP and larger phytoflagellates are actively mixotrophic or not has important implications for both marine food webs and biogeochemical cycling (Mitra et al., 2013, 2014; Ward and Follows, 2016). As an important example for marine fisheries, the predominance of mixotrophy over strict phototrophy appears to enhance the transfer of biomass to higher levels in the food chain, leading to larger mean organism sizes (Ward and Follows, 2016). Current changes in sea water temperatures are thought to be causing shifts in SP communities, with largely unknown consequences for the magnitude of active mixotrophy (Wilken et al., 2013; Ward, 2015). Despite this cumulative evidence for their significance, studies assessing marine mixotrophy *in situ* remain relatively scarce, and mixotrophic SP have to date been often excluded from biogeochemical and food web models (Flynn et al., 2013).

One problematic aspect for the correct parametrization of mixotrophic SP bacterivory is that we still lack a good understanding of how different environmental parameters influence SP feeding in marine systems. It is thought that the primary triggers for feeding among bacterivorous SP are the availability of nutrients, prey concentration and size, and/or irradiance levels (Jones, 2000). However, the regulatory effect and interplay between these factors is complex, differing strongly between tested species. For example, light has been shown to strongly influence SP feeding rates both positively (Caron et al., 1993; Brutemark and Granéli, 2011) and negatively (McKie-Krisberg et al., 2015). Additionally, there is an energetic and genomic cost to maintaining both photosynthetic and phagotrophic machineries in a single organism, which can lead to a strict control of when and how much SP feed (Raven, 1997). Thus, both SP feeding rates and percentages of actively feeding mixotrophic SP (AMSP) could shift significantly over even small temporal and spatial scales. In fact, in freshwater systems, studies have shown significant shifts in SP community bacterivory on small spatial scales related to plant coverage (Izaguirre et al., 2012) and strong shifts in the bacterivory of specific phylogenetic groups along the diel cycle (Urabe et al., 2000; Pålsson and Granéli, 2003). Nonetheless, it remains to be tested whether such shifts can generally be observed for the whole SP community in freshwater and marine systems and how this could impact the way we measure AMSP bacterivory.

SP bacterivory is predominantly measured using techniques adapted and optimized for determining bacterivory rates in heterotrophic nanoflagellates (HNF), especially the use of surrogate prey (e.g., see review table within Unrein et al., 2007). In principle, this is a valid approach, since the mechanisms for finding, capturing, ingesting and digesting prey in SP, though diverse, are thought to be comparable to those of HNF. However, a recent review by Weisse et al. (2016) addressed the fact that potential biases could arise from treating bacterivory in SP as equivalent to that of HNF, aside from those inherent to the well-known limitations for these methods (Landry et al., 1991; Vaque et al., 1994). Specifically, two routinely employed central assumptions of these methods were highlighted: (1) bacterivory will remain constant over ‘short’ time scales, enabling the extrapolation of measured hourly bacterivory rates to, e.g., daily bacterivory rates; and (2) community ingestion rates (determined as bacterivory rate divided by the total abundance of the studied protist group) can be assumed to approximate the average ingestion rate of all feeding individuals. To be realistic, this latter assumption requires that a significant proportion of the studied protist community be feeding. Adopting both these assumptions ignores the fact that the regulation of SP feeding has a much tighter coupling to environmental parameters than that of HNF, and that most SP do not need to feed if the abiotic environment meets their energetic and nutritional requirements.

In the present case study, the potential biases introduced by adopting the assumptions outlined in Weisse et al. (2016) and in the discussion above were tested at three marine coastal stations during winter and summer. A combination of methods to determine bacterivory rates [fluorescently labeled bacteria (FLB) used as a surrogate prey] and the percentage of AMSP (acidotropic probes employed as food vacuole markers) were applied at different time points along the diel cycle. We determined whether sampling at different times of day lead to significant differences in determined SP feeding rates, relative bacterivorous impact of different SP groups, and their perceived relative importance when compared to HNF. In addition, the feasibility of applying acidotropic probes to measure levels of AMSP in the field was tested for the first time.

## Materials and Methods

### Sampling

Sampling was conducted at Helsingør North harbor on the 18th of December 2015 and the 31st of May 2016 at two stations located outside [station 1 (st. 1)] and inside the harbor walls [station 2 (st. 2)]; and on the 9th of June 2016 at station 3 [inside the harbor walls (st. 3)] (**Table 1**). Sampling time points were selected to cover different phases of the photoperiod. In all cases, measurements of irradiance and temperature were taken 1 cm below the water surface with an ULM-500 light meter (Walz GmbH, Germany) and a digital thermometer respectively. At every time point, 40 μm – filtered surface seawater was stored in the dark and on ice for subsequent analysis of the fraction of AMSP using acidotropic probes (see below; in December a single sample was collected for each time point and study site, while in May and June, triplicate samples were taken). In parallel, at selected time points in December 2015 and May 2016 experiments to measure bacterivory rates were conducted at station 1 as described below (**Table 1**).

**Table 1:** Summary of sampling times for food vacuole marker assays in stations 1 and 2 (December 2015 and May 2016) and station 3 (June 2016), including water temperature and surface water irradiance values.

	Pre-twilight	Morning		Mid-day		Afternoon		After dusk
**December**								
Sampling time	07:00	10:00^∗^		n/a		14:00^∗^		16:00^∗^
Weather conditions	Overcast and wind-still					Overcast and wind-still		
Water temperature (°C) st. 1/st. 2	7.6/7.6	7.0/7.0				7.0/7.0		7.2/7.2
Irradiance (μE m^-2^ s^-1^) (determined at st. 1)	0	35				25		0
**May**								
Sampling time	03:00^∗^	10:00		13:00^∗^		17:00^∗^		23:00
Weather conditions	Partially cloudy	Partially cloudy		Overcast rain		Partially cloudy		Partially cloudy
Water temperature (°C) st. 1/st. 2	14.6/16.8	15.6/17.0		17.0/17.4		16/18.0		15.5/17.1
Irradiance (μE m^-2^ s^-1^) st. 1/st. 2	0/0	850/650		250/190		540/470		0/0
**June**								
Sampling time	n/a	07:45	09:15	10:45	12:15	13:45	15:00	n/a
Weather conditions		Sunny and wind-still						
Water temperature (°C) st. 3		16.7	17	17	17.6	18.2	18.4	
Irradiance (μE m^-2^ s^-1^) st. 3		630	900	1220	1200	1400	900	

*Time points are indicated (^∗^) were FLB experiments were carried out in concert at station 1 in December 2015 and May 2016. Shading indicates night-time sampling points.*

*n/a: not applicable, no measurement was taken.*

### Determination of Bacterivory Rates

Bacterivory rates for both SP and HNF were studied by measuring uptake of FLB in 40-min incubations using a slightly modified version of the protocol introduced by Sherr et al. (1987). The present study employed a 1:1 mix of *Brevundimonas diminuta* and *Photobacterium angustum* (strain S14) converted to FLB as described by Vazquez-Dominguez et al. (1999). The *P. angustum* strain was harvested during the starvation phase in order to obtain smaller cells, as described in Anderson et al. (2011). Thus, both strains were within the size range observed for the natural community of the Øresund (visual observations with an in-built ocular ruler on the microscope). The incubation time of 40 min was selected from the linear uptake phase of an 80 min time series, with samples taken every 20 min, conducted prior to this study. The incubations were carried out in custom built 50 L incubation chambers filled with surface seawater. These were equipped with holders which allowed the incubation bottles to be secured upside down to the bottom of the chamber, to avoid the lid of the bottles casting a shadow. Water temperature in the incubation chambers was monitored with a digital thermometer, and ice or hot water was added as necessary to maintain the *in situ* temperature. An ULM-500 light meter was used to monitor shifts in irradiance.

To start the experiment, surface water was collected, pre-filtered through a 40 μm mesh and dispensed into triplicate 250 mL glass bottles, which were immediately inoculated with FLB to a concentration of 10–15% of *in situ* bacterial abundance. 50 mL samples were taken at time 0 and 40 min and fixed v/v with 4% very cold glutaraldehyde (diluted from a 25% stock with sterile filtered sea water) (Sanders et al., 1989). Samples were stored cool and in the dark for a minimum of 2 h and a maximum of 24 h. Subsequently, for each sample, 4 mL were filtered on to 0.2 μm black Cyclopore polycarbonate filters for bacterial quantification, 20 mL on to 0.8 μm black polycarbonate filters for flagellates <5 μm in size, and 50 mL on to 3 μm white polycarbonate filters for flagellates 5–20 μm in size (all Whatman, GE Healthcare Europe GmbH). All samples were stained for 2 min with a 0.01 mg/mL solution of 4′,6-diamidino-2-phenylindole (DAPI) before mounting for epifluorescence microscopy. Samples were quantified under a BX-50 epifluorescence microscope at 1000X using filter sets U-FUW for DAPI, U-FGW for phycoerythrin autofluorescence, and U-FBNA for chlorophyll autofluorescence and FLB fluorescence (all Olympus, Co., Japan). Pigment fluorescence was used to distinguish between HNF (no pigments), cryptophytes (phycoerythrin fluorescence; Cry-SP) and other SP (chlorophyll but no detectable phycoerythrin fluorescence; NCry-SP). Size was further used to distinguish a total of 8 protist groups (**Table 2**). For each group, a minimum of 200 cells or 150 counting fields were quantified and examined for the presence of ingested FLB.

**Table 2:** SP groups distinguished via microscopy and flow cytometry in December 2015, May 2016, and June 2016.

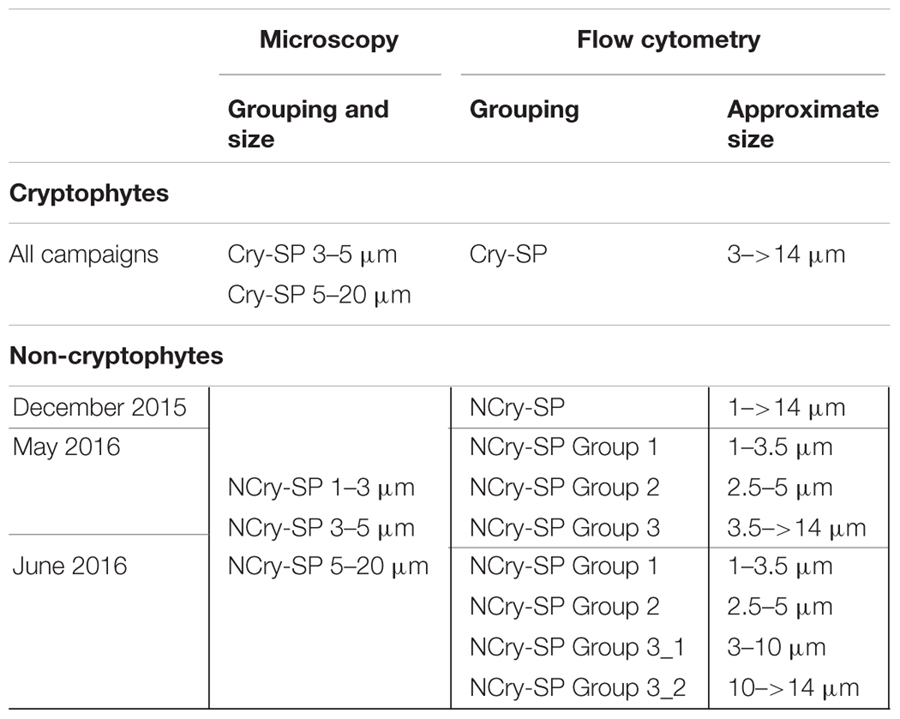

The grazing rate (G) for the whole SP and HNF community and each flagellate group distinguished was determined at each sampling time point as:

$$\begin{array}{c} G\;=\;((I_{40}{-I}_{0})\;\times \;B/F)/t \end{array}$$

where I is the total number of ingested FLB at t40 and t0, B is the natural bacterial abundance, F is the inoculated FLB abundance for that depth and t is the incubation time (in h). Daily bacterivory rates were estimated by multiplying hourly rates by 24, and these values were then used to estimate the percentage of bacterial standing stocks consumed per day. Ingestion rates for total HNF, SP and AMSP were determined dividing the respective G by the total abundance for that protist group. AMSP abundance was estimated by multiplying SP abundance by the percentage of mixotrophically active cells as determined with acidotropic probes (see below).

### Determination of the Percentage of AMSP

The percentage of SP cells containing food vacuoles, and therefore assumed to be actively feeding, was determined using the acidotropic probe LysoTracker Green DND-26 (LyT G) (ThermoFisher Scientific) according to the protocol from Sintes and del Giorgio (2010). Briefly, samples were incubated with a final concentration of 50 nM LyTG for 3 min and measured for 5–7 min on a FACS Canto II flow cytometer (BD Biosciences) using a low flow rate. TrueCount beads (BD Biosciences) were used to determine the flow cytometer flow rate, while beads of known sizes were used to estimate protist size. Between 2 and 5 groups of SP could be detected based on differences in forward scatter (FSC; proxy for cell size), side scatter (SSC; proxy for cell complexity) and pigment fluorescence (chlorophyll and phycoerythrin) (**Table 2** and Supplementary Figure 1). One cryptophyte group was distinguished (Cry-SP, high phycoerythrin levels relative to chlorophyll fluorescence) and 1–4 non-cryptophyte groups (NCry-SP; low phycoerythrin levels relative to chlorophyll fluorescence). These groups are similar but not directly comparable to those distinguished via microscopy due to differing size ranges. Large cyanobacteria were discriminated from the smallest protists based on their relatively high phycoerythrin to low chlorophyll content and differences in SSC.

To determine the percentage of cells with labeled food vacuoles, first a sample with no added LyTG was measured on the flow cytometer and the baseline green fluorescence for each SP group was determined (Supplementary Figure 2). Cells that exhibited a higher green fluorescence after incubation with LyTG were considered to have labeled food vacuoles, and their percentage with respect to the total abundance of that SP group was calculated. All measurements were duplicated, and percentages were only calculated if more than 30 cells were detected for a given group (bellow this cell count, duplicate measurements became highly variable).

Prior to the experiments the potential for LyTG to stain other acidic organelles, such as chloroplasts, was tested with 12 phylogenetically diverse SP strains grown under nutrient and light replete conditions, favorable for a strictly photosynthetic lifestyle. All species are known mixotrophs or are assumed to have the potential for mixotrophy based on studies with closely related SP (e.g., McKie-Krisberg et al., 2015): the chlorophytes *Pyramimonas mitra* K-0241, *Pyramimonas disomata* K-0285, *Pyramimonas melkonianii* K-0628, *Mantoniella* spp. K-0284 and K-1106, *Nephroselmis rotunda* K-0251 and *Nephroselmis pyriformis* K-0557, the haptophytes *Chrysochromulina simplex* K-0272, *Chrysochromulina brevifilum* K-0560 and *Imantonia* sp. K-0624 and the cryptophytes *Teleaulax acuta, Teleaulax amphioxeia* and *Geminigera cryophila*. All measurements were conducted as described above. All cultures were obtained from the Scandinavian Culture Collection of Algae and Protozoa (SCCAP; now transferred to NIVA, Oslo, Norway), with the exception of the three cryptophyte strains which were isolated by PJH and are maintained at MBS.

## Results

### Study Site Characteristics

The present case study focused on three stations and three different dates in December 2015 and May and June 2016 (**Table 1**). In December, water temperature was constant between the three sampling time points and surface water irradiance was overall very low. In May, water temperature was more variable and surface water irradiance shifted strongly between the day-time sampling points due to shifts in weather conditions. The June 2016 campaign was carried out 1 week after the May 2015 sampling and focused on a more sheltered sampling site, st. 3, during stable weather conditions.

### Application of Acidotropic Probes to Measure *In Situ* Levels of AMSP

**FIGURE 1:**
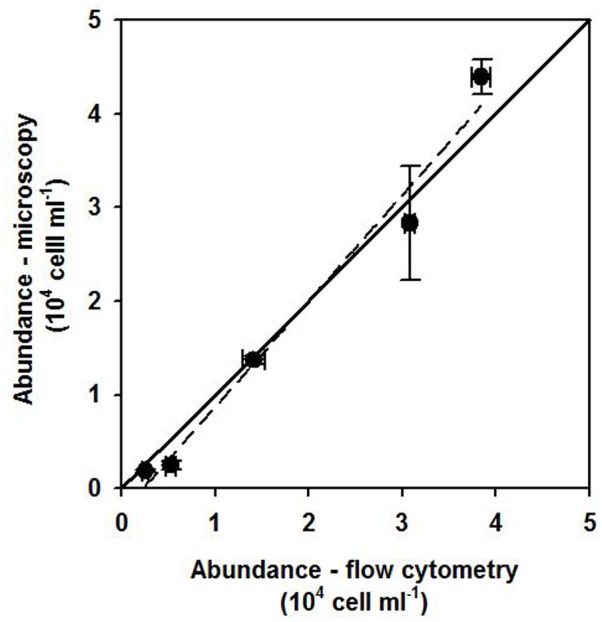
Comparison between flow cytometry and microscopy SP counts for time points were FLB experiments were carried out at station 1 in December 2015 and May 2016 The solid line indicates a 1:1 ratio and the dashed blacked line the linear regression for the data points (*R*^2^: 0.99).

Prior to sampling, tests were carried out with 12 phylogenetically diverse cultured SP strains to detect potential unspecific binding of the dye. No measurable LyTG staining could be observed for any strain when grown under conditions favoring a strictly phototroph lifestyle (0% of cells with LyTG staining above the threshold after subtracting blank values; see example in Supplementary Figure 2). In flow cytometric measurements of field samples, SP could always be clearly distinguished from background events, and a cross comparison to SP abundance determined microscopically at selected time points in st. 1 closely approximated the 1:1 line (**Figure 1**).

**FIGURE 2:**
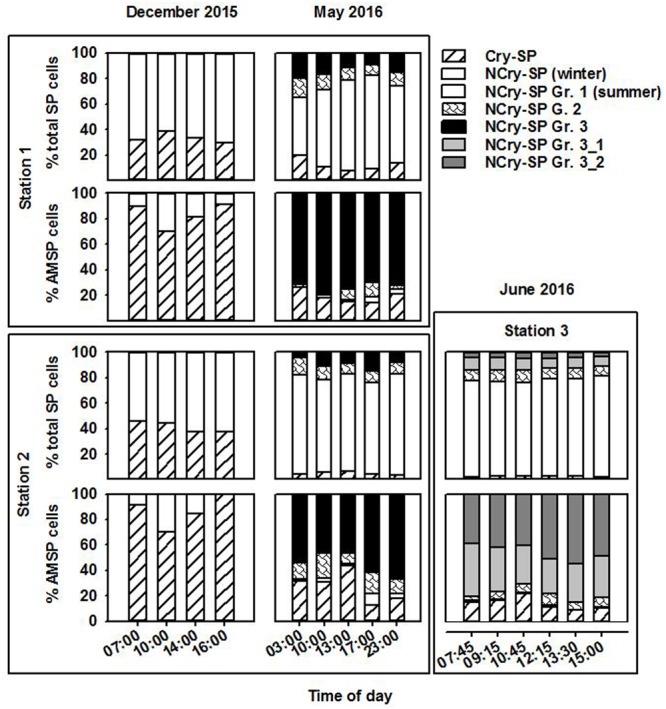
Relative contribution of the different groups (Gr.) distinguished on the flow cytometer to the total SP and AMSP pool at station 1, station 2, and station 3 in December 2015, May 2016, and June 2016.

In December, SP were only differentiated into Cry-SP and NCry-SP (Supplementary Figure 1 and **Table 2**; it should be noted that during this sampling only duplicate LyTG measurements were taken and statistical comparisons of the data are not possible). Both stations (1 and 2) were similar (**Figure 2** and **Tables 1, 3**). NCry-SP were more abundant than Cry-SP (**Figure 2**) and overall smaller, with the majority of recorded cells ranging around an estimated 1–3 μm in size vs. 3–7 μm, respectively (Supplementary Figure 1). Percentages of NCry-AMSP were low and relatively constant between time points and stations (range: 1–7% of total NCry-SP). Percentages of Cry-AMSP were higher at all time points with maximum values found during the night (night and day ranges of 36–76% and 18–32%, respectively in st. 1, and 26–28% and 9–25%, respectively in st. 2). Overall, NCry-SP were more important to the total SP pool in terms of abundance, but Cry-SP dominated the AMSP pool at all time points and sampling stations (**Figure 2**).

**Table 3:** Average SP abundance and percentage of actively feeding mixotrophic SP cells (AMSP) measured in winter and summer at all three sampling stations (standard deviation in parenthesis).

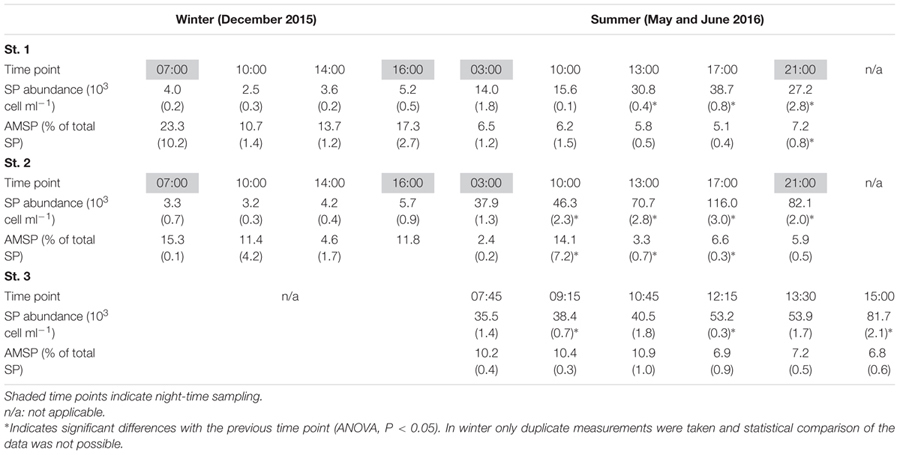

**FIGURE 3:**
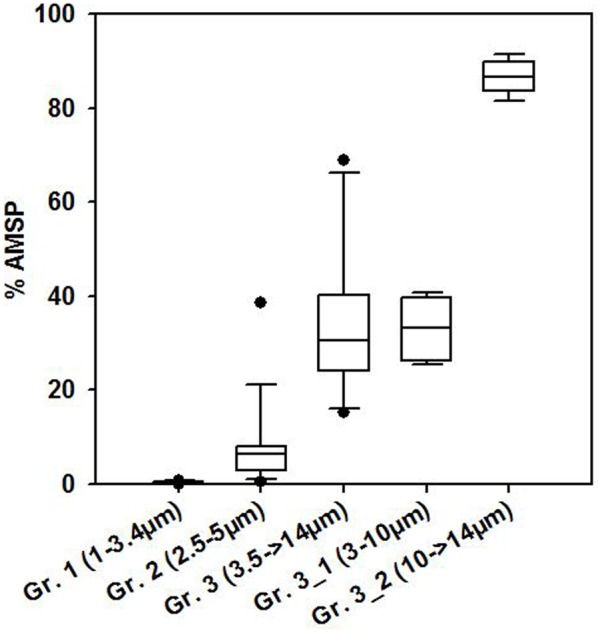
Boxplot showing average percentages of AMSP for the different NCry-SP groups (Gr.) distinguished on the flow cytometer in summer. The data was pooled for all time points sampled at all three study sites during both summer sampling dates.

The May and June campaigns were conducted 1 week apart, centering respectively on st. 1 and st. 2, and st. 3 (**Table 1**). Total SP abundance was higher than in winter and tended to significantly increase as the day progressed in all sampling stations, followed by a decrease after dusk in st. 1 and st. 2 (**Table 3**; ANOVA, *P* < 0.05). In contrast, percentages of total AMSP were overall lower than in winter and did not show any clear temporal variations (**Table 3**). In terms of SP subgroups detected, the Cry-SP grouping remained the same as in winter, but NCry-SP could be separated into three groups in st. 1 and st. 2 and four groups in st. 3 (Supplementary Figure 2 and **Table 2**). Cry-SP showed strong and significant differences in abundance (all stations) and percentages of AMSP (st. 2 and st. 3) (**Figure 2**; ANOVA, *P* < 0.05). However, in contrast to winter, the highest levels of Cry-SP AMSP were found inside the harbor (st. 2 and st. 3) in the morning hours (range of 66–73% of AMSP measured at 10:00 in st. 2 and between 7:45 and 10:45 in st. 3, compared to a range 8–30% of AMSP measured at all time points in st. 1, and in the afternoon and at night in st. 2 and st. 3 (03:00, 13:00–23:00 in st. 2 and 12:15–15:00 in st. 3). NCry-SP Group 1 dominated the SP pool in terms of abundance at all three stations (**Figure 2**). Their levels of AMSP, in contrast, were very low (**Figures 2, 3**; 0–1% of cell-counts for this group). However, it should be noted that due to their very high abundance this would still imply abundances of up to 10^3^ NCry-AMSP Group 1 cells ml^-1^. NCry-SP Group 2 was generally more abundant at st. 2 and st. 3 than st. 1, but were overall marginal constituents of both the SP and AMSP pools (**Figure 2**). NCry-SP Group 3 was a relatively marginal constituent of total SP pools in terms of abundance but dominated the AMSP pool at all summer stations and sampling time points (**Figure 2**). In st. 3, where this group could be further divided into two subgroups, NCry-SP Group 3_2 appeared to be a composed almost entirely of mixotrophically active cells at all time points tested (**Figure 3**). Overall, for NCry-SP there was a clear trend in summer toward increased percentages of AMSP with cell size (**Figure 3**).

### Bacterivory Rate Measurements

**FIGURE 4:**
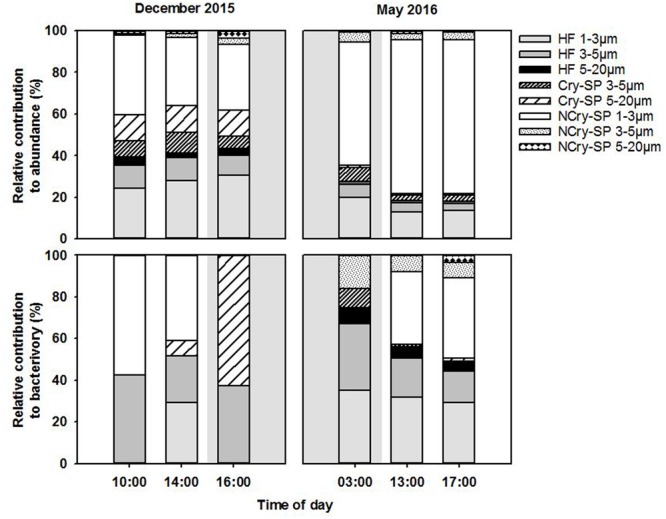
Relative contribution to total flagellate abundance and to total bacterivory rates of the different protist groups distinguished microscopically in December 2015 and May 2016. Shaded background areas indicate night-time sampling.

Fluorescently labeled bacteria addition experiments were carried out at st. 1 in December 2015 and in May 2016, covering in each case 2 day-time and one night-time sampling time points (**Table 1**). Total SP and HNF cell-counts remained low and constant along the diel cycle in December and were significantly higher and more variable in May (**Table 4**). Bacterial abundance showed only small differences between sampling dates and, within each date, presented relatively small but significant shifts during the diel cycle (**Table 4**; ANOVA, *P* < 0.05). Size class compositions of SP and HNF were highly similar for the different time points of both study dates but differed between the study dates (**Figure 4**). HNF were dominated by cells 1–3 μm in diameter, and SP by NCry-SP 1–3 μm in diameter. In December, Cry-SP also had a high relative importance in terms of abundance, which strongly decreased in May.

**Table 4:** Average bacterial abundance and total HNF and SP abundance, measured hourly bacterivory rates and estimated daily bacterivory rates for December 2015 and May 2016 (standard deviation in parenthesis).

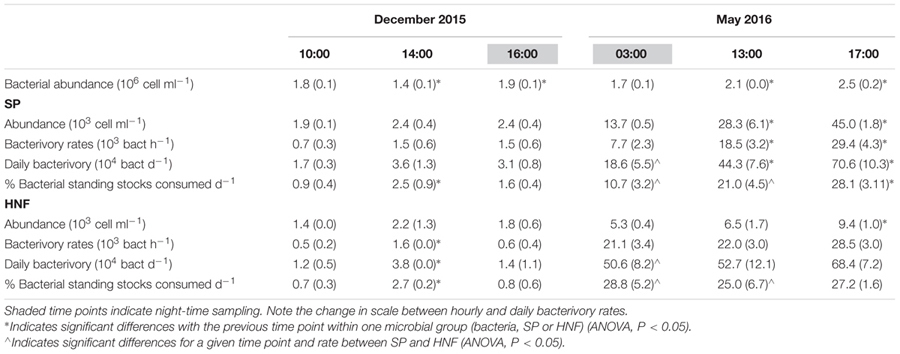

Calculated hourly bacterivory rates for total SP were relatively constant in December (**Table 4**; ANOVA, *P* > 0.05), but strongly and significantly shifted between time points in May (ANOVA, *P* < 0.05). This resulted in stable estimated daily bacterivory rates in December, but up to an almost fourfold difference in daily rates in May depending on which time point was used for calculations. The percentage of bacterial standing stocks consumed daily, which also takes into account shifts in bacterial abundance, showed less variation than daily bacterivory rates in May, but showed significant differences in December (**Table 4**; ANOVA, *P* < 0.05). Additionally, for both sampling dates, the primary group of bacterivores among the SP changed drastically at the different time points analyzed (**Figure 4**). NCry-SP 1–3 μm in diameter dominated bacterivory during day-time on both sampling dates, but feeding rates were close to or below the detection limit of the method at night (76–82% and 0–3% of total SP bacterivory respectively). In winter, SP night-time feeding was dominated by Cry-SP 5–20 μm in diameter, while in summer it was carried out by Cry-SP and NCry-SP 3–5 μm in diameter.

Heterotrophic nanoflagellate bacterivory remained overall relatively constant during the diel cycle on both study dates, both in terms of hourly rates (**Table 4**) and the relative bacterivorous importance of different HNF groups (**Figure 4**). The exception was 14:00 in December, which showed a slight but significant increase in bacterivory rates (ANOVA, *P* < 0.05), accompanied by a strong increase in the relative bacterivorous importance of HNF 1–3 μm in diameter (**Figure 4**; 57% of total HNF bacterivory compared to 0% at the other two time points). Estimated daily bacterivory rates and percentages of bacterial standing stocks consumed daily remained relatively constant on both sampling dates regardless of which time point was used for calculation (**Table 4**). The relative importance of HNF as bacterivores when compared to SP was constant in December, but in May was much higher at night-time than during day-time (75% of total bacterivory vs. an average of 52%; **Figure 4** and **Table 4**).

Ingestion rates calculated for total SP, estimated total AMSP and total HNF remained constant along the diel cycle on both study dates for all groups (**Table 5**; ANOVA, *P* > 0.05). However, ingestion rates for the total SP community were always significantly lower than AMSP ingestion rates (ANOVA, *P* < 0.05), reaching up to a 16-fold difference in May. This difference also impacted the comparison between seasons and between SP and HNF. SP ingestion rates remained constant between December and May (ANOVA, *P* > 0.05) and were equal (December; ANOVA, *P* > 0.05) or lower (May; ANOVA, *P* < 0.05) than HNF ingestion rates. In contrast, AMSP ingestion rates were significantly higher in May than in December (ANOVA, *P* < 0.05) and were consistently significantly higher than HNF ingestion rates (**Table 5**).

**Table 5:** Average ingestion rates (bacteria individual^-1^ h^-1^) for total SP, estimated AMSP and total HNF for December 2015 and May 2016 (standard deviation in parenthesis).

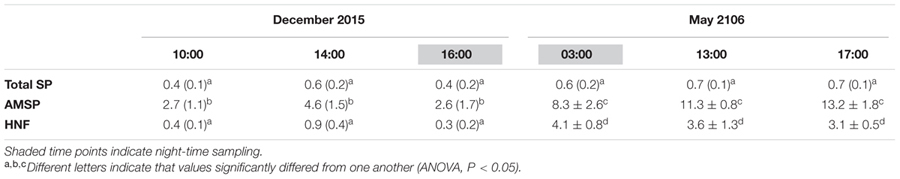

## Discussion

### Combined Use of Acidotropic Probes and Surrogate Prey to Measure Mixotrophy in the Field – Methodological Considerations

Acidotropic probes, such as LyTG, have been proposed over the last decade as ideal tools for a fast and effective detection of food vacuoles among protists, enabling the distinction between feeding and non-feeding constituents of a population or community (Rose et al., 2004; Carvalho and Granéli, 2006; Sintes and del Giorgio, 2010). However, their experimental application has remained very limited (Vázquez-Domínguez et al., 2005; Carvalho and Granéli, 2010; Sintes and del Giorgio, 2010), and this is the first time they have been applied in the field to detect mixotrophy. One major constraint has been the possibility of unspecific binding of acidotropic probes to acidic organelles other than food vacuoles, such as chloroplasts. However, this remains untested for SP. In the present study, no unspecific binding was observed in 12 phylogenetically diverse SP strains when grown under conditions favorable for strictly phototrophic growth. We therefore conclude that with the method employed and for the phytoflagellate size range tested, there is no detectable effect of unspecific binding on the quantification of actively feeding SP.

A primary advantage of LyTG is that it allows a very fast and direct assessment of what fraction of a population is actively feeding, making it a powerful supplementary tool to classical methods for determining bacterivory rates, such as the use of surrogate prey. However, care should be taken when comparing the two methods for the following reasons: (1) LyTG will likely detect a broader range of AMSP than the FLB method. This is due both to the specificity of the method (the FLB approach is specific for bacterivory, while LyTG staining does not discriminate between prey type) and the well-known problematic of certain protist groups not feeding on FLB (Landry et al., 1991). (2) While the FLB method offers a punctual measurement of feeding rates at the time of sampling, measurements with LyTG will also reflect the ‘short’-term feeding history of the protist community. This is due to the fact that the method does not discriminate between ‘new’ and ‘old’ food vacuoles, and protists employ variable lengths of time for digestion, ranging from minutes to hours (Gonzalez et al., 1990; Boenigk et al., 2001). And (3) SP groups distinguished by flow cytometry and microscopy are not always directly comparable, since the flow cytometer provides a more nuanced and fluid grouping of SP based on size and pigment fluorescence. This can be exemplified in the present study with the microscopically determined group NCry-SP 1–3 μm, which includes cells from the flow cytometrically determined NCry-SP Group 1, Group 2 and, occasionally, Group 3 (**Table 2**). However, despite these differences, the principal patterns observed in this study remained constant between methods, indicating that their combined application provided a robust and highly complementary view of mixotrophy in the selected study systems.

### Case Study Results: Is Our View of *In Situ* Bacterivory by Mixotrophic Phytoflagellates Biased by Methodological Considerations?

In the present case study two central assumptions routinely employed by methods to determine bacterivory were tested for their applicability to SP. Namely: (A) bacterivory for the whole SP community and distinct SP groups will remain constant over ‘short’ time scales (hours to a day); and (B) community ingestion rates can be assumed to approximate the average ingestion rate of all feeding individuals. The results obtained in the present case study indicate that neither of the assumptions could be reliably applied to SP without a significant potential bias.

#### Shifts in Bacterivory over Short Time Scales

Total SP hourly bacterivory rates significantly differed between tested time points during the summer campaign. This lead to up to a fourfold difference in estimated daily bacterivory rates, and a threefold difference in the percentage of bacterial standing stocks consumed daily depending on the time point used for calculation (**Table 4**). These strong differences also impacted the perceived relative importance as grazers of HNF and SP, ranging from being equally important during day-time sampling points to the dominance of HNF at night (∼75% of total bacterivory) (**Figure 4**). In winter, when SP abundance and feeding rates were considerably lower, bacterivory rates remained constant, but significant differences could still be observed in the percentage of standing stocks consumed daily depending on the time point used for calculation. Differences in bacterivory along the diel cycle have been observed pigmented flagellates feeding on *Synechococcus* (An-Yi et al., 2009). However, to our knowledge, this is the first study testing and demonstrating that sampling at different times of day can significantly bias the perceived bacterivorous impact of SP on the whole bacterial community.

An additional consideration of note is that the relative importance of different SP groups as bacterivores also differed very strongly between sampling time points in both winter and summer. As an example, in winter, in the space of 2 h, bacterivory shifted from being carried out almost entirely by NCry-SP 1–3 μm in diameter (14:00; day) to being exclusively carried out by Cry-SP (16:00; night), without any significant shift in total SP bacterivory rates (**Figure 4** and **Table 4**). Significant shifts in feeding rates along the diel cycle for specific SP groups have also been observed for other systems, such as for *Cryptomonas* spp. and *Dinobryon* spp. in lakes (Urabe et al., 2000; Pålsson and Granéli, 2003), and can be as strong as the differences in bacterivory rates found between seasons in longer term studies (Roberts and Laybourn-Parry, 1999; Unrein et al., 2013). It is additionally important to note that in the present study these strong shifts were at times masked by constant total SP bacterivory and ingestion rates (**Tables 4, 5**). These constant rates did not reflect a stable system, but rather very strong shifts in major bacterivorous SP groups, with different abundances and ingestion rates.

Potential reasons for group specific shifts in bacterivorous importance could be the influence of irradiance on feeding (Caron et al., 1993; Brutemark and Granéli, 2011; Izaguirre et al., 2012), diel shifts in prey activity (Gasol et al., 1998), potential SP migration patterns (Olli, 1999; Shikata et al., 2015), and/or SP phased cell cycles, with coordinated cell division (Weiler and Eppley, 1979; Jacquet et al., 2001). In the present study, since we did not confine a single water mass and sample it throughout the day, we cannot exclude shifting water masses as a partial reason for our results. However, the results obtained were highly consistent between seasons and in comparison to other studies, pointing to the urgent need for further studies on the influence of diel cycles on SP ecology. In particular, we wish to highlight the strong relationship observed between the diel cycle and the feeding of pico-phytoplankton and cryptophytes, with emphasis on its potential impact on estimates of the global contribution to bacterivory for these groups.

Finally, HNF bacterivory remained relatively constant throughout the diel cycle in the present study during both seasons. This is consistent with results obtained in other systems (Marrase et al., 1992; Cuevas and Morales, 2006) and would appear to support the feasibility of converting hourly to daily rates for HNF. However, there are indications that HNF bacterivory can also be subject to diel cycles (Wikner and Rassoulzadegan, 1990; Ng and Liu, 2016), especially when consuming phototrophic prey (Dolan and Šimek, 1999), pointing to the necessity for further studies in this field.

#### SP vs. AMSP Ingestion Rates and Percentages of AMSP

In the present study, using the total SP or the estimated AMSP abundance for calculating ingestion rates lead to two significantly different views of mixotrophy in the study system. Ingestion rates determined employing total SP abundance were low, within the range recorded in other systems for the whole SP community (Unrein et al., 2007; Zubkov and Tarran, 2008). They additionally did not change between seasons and were comparable to, or lower than, HNF ingestion rates. Combined with protist abundance and bacterivory rates, this provides an overall impression of a system where SP feed at low and constant rates, but can have still a strong impact on the bacterial community due to their high abundance. However, restricting the calculation of ingestion rates to AMSP lead to rates that were up to 16-fold higher than those determined for SP, significantly differed between summer and winter and were significantly higher than HNF ingestion rates. This provides an opposing view of the same system, where only a small fraction of SP are actively feeding, but their high bacterivory rates can lead to a strong impact on the bacterial community. The AMSP abundance employed here can only be considered an estimate, since for reasons outlined earlier in the discussion percentages of mixotrophically active cells determined via LyTG might overestimate the number of active bacterivores. However, the AMSP ingestion rates obtained are in the range of those from studies which focused on the feeding of specific SP species or groups known to be active bacterivores (Domaizon et al., 2003; Pålsson and Daniel, 2004; Unrein et al., 2013). In addition, a high fraction of non-feeding SP is consistent with the notion of a strict environmental regulation of feeding in SP. The results obtained thus indicate that caution should be exercised drawing conclusions from SP ingestion rates when the proportions of actively feeding cells are unknown.

Comparing SP and AMSP ingestion rates for the sub-groups distinguished in the present study is harder, since there is not a direct correspondence between the groupings established by flow cytometry and microscopy (see discussion above). However, overall a marked increase could be discerned with SP size in the percentage of AMSP (**Figure 3**) and observed frequency and number of ingested FLB (data not shown). Cells in the picoeukaryote range (corresponding roughly to NCry-SP Groups 1 and 2) were predominantly strictly phototrophic at the time of sampling. In contrast, the largest SP group distinguished on the flow cytometer (NCry-SP Group 3_2) was composed almost exclusively of mixotrophic cells. This pattern probably reflects the differing energetic and nutritional requirements of the different size classes, with larger cells finding it harder to meet their needs solely from the abiotic environment. Whether this is a generalized pattern present in other environments is another field of interest for future studies. Increasing water temperatures appear to be leading toward smaller phytoplankton cells sizes (Li et al., 2009; Sato et al., 2015) with largely unknown consequences for the phototrophy/mixotrophy balance.

## Conclusion

Overall, results from the present case study indicate that methodological considerations can considerably bias our view of *in situ* mixotrophy: (1) Disregarding the influence of the diel cycle on SP can lead to erroneous estimates of both SP daily bacterivorous impact and their relative importance as bacterivores compared to HNF. It can additionally bias our view of which SP groups are the principal bacterivores in a given system. And (2) disregarding the possibility that a significant fraction of the SP community may not be feeding can significantly bias ingestion rates and may considerably alter our perception of mixotrophy in a given system. We wish to stress that this is a case study conducted in a dynamic coastal zone, so our results cannot be directly extrapolated to other systems. However, the present study points to several worrisome aspects that merit further study in other environments. Overall, we recommend that (a) care should be taken extrapolating punctual measurements of bacterivory in SP to longer time scales. Where possible different times of day should be sampled and longer term studies should try to consistently sample at same time of day; (b) determining which the principal SP bacterivores are in a given system or the relative role of SP and HNF should not be based on punctual measurements; and (c) conclusions based on community ingestion rates for SP should be treated with caution when the levels of actively feeding cells are not known. The application of LyTG in concert to classic techniques is a simple and rapid technique to correct this latter problem.

## Author Contributions

RA and PH designed and conducted the experiments, RA analyzed samples and data; KJ provided advice and method training; RA, PH, and KJ were involved in manuscript preparation.

## Conflict of Interest Statement

The authors declare that the research was conducted in the absence of any commercial or financial relationships that could be construed as a potential conflict of interest.
