# Supplementary material for: Mixotrophic Phytoflagellate Bacterivory Field Measurements Strongly Biased by Standard Approaches: A Case Study
Source: Front Microbiol. 2017 Jul 26;8:1398. doi: 10.3389/fmicb.2017.01398 (PMC5526857; doi:10.3389/fmicb.2017.01398)

Supplementary figure 1. SP groups distinguished in December in st. 1 and 2 (A), st. 1 in May (B), st. 2 in May (C) and st. 3 in June (D). Red events are Cry-SP, green events represent total NCry-SP in December (A) and NCry-SP Group 1 in May and June (B, C and D), yellow events are NCry-SP Group 2 and blue events are NCry-SP Group 3. In June (D) blue events are further divided into Group 3_1 (light blue) and Group 3_2 (dark blue). Black events at the bottom of the scattogram represent the location of beads of known sizes: respectively, 2, 3.4, 5.11, 7.4, 10.3, 14.7 µm in size. It should be noted that scale-bars are arbitrary. Background events have been removed from these scattograms for clarity.

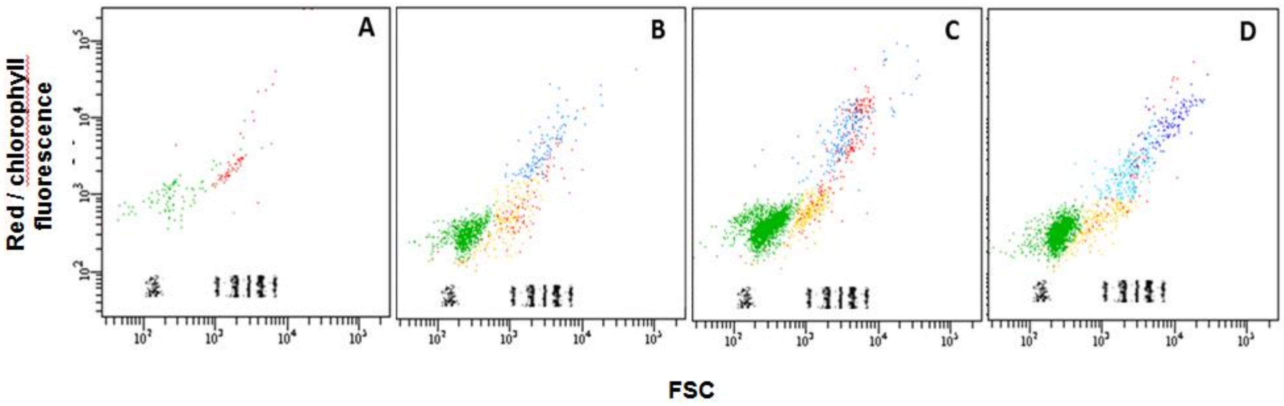

Supplement: Supplementary file 1 [file Image_1.PDF]
