# Supplementary material for: Mixotrophic Phytoflagellate Bacterivory Field Measurements Strongly Biased by Standard Approaches: A Case Study
Source: Front Microbiol. 2017 Jul 26;8:1398. doi: 10.3389/fmicb.2017.01398 (PMC5526857; doi:10.3389/fmicb.2017.01398)

Supplementary figure 2. Examples of positive and negative LyTG staining detection for cultures (A, B, C) and protist groups in natural samples (D, E, F). Detection of *Pyramimonas disomata* cells (A; red events) and the corresponding yellow/green fluorescence before (B) and after (C) staining with LyT G. Detection of Cry-SP in May 2016 (D; red events) and the corresponding yellow/green fluorescence before (E) and after (F) staining with LyT G.

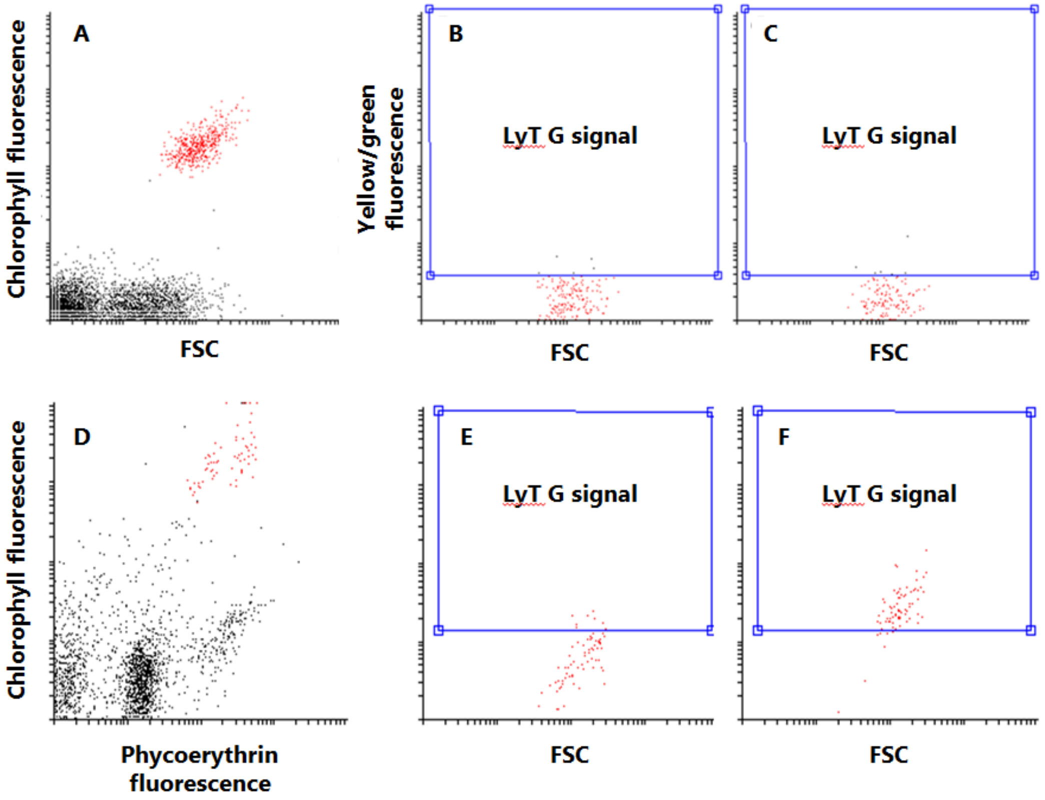

Supplement: Supplementary file 2 [file Image_2.PDF]
